# MArVD2: a machine learning enhanced tool to discriminate between archaeal and bacterial viruses in viral datasets

**DOI:** 10.1038/s43705-023-00295-9

**Published:** 2023-08-24

**Authors:** Dean Vik, Benjamin Bolduc, Simon Roux, Christine L. Sun, Akbar Adjie Pratama, Mart Krupovic, Matthew B. Sullivan

**Affiliations:** 1 Department of Microbiology, The Ohio State University, Columbus, OH 43210 USA; 2 Center of Microbiome Science, The Ohio State University, Columbus, OH USA; 3 DOE Joint Genome Institute, Lawrence Berkeley National Laboratory, Berkeley, CA USA; 4 Archaeal Virology Unit, Institut Pasteur, Université Paris Cité, CNRS UMR6047, Paris, France; 5 Department of Civil, Environmental and Geodetic Engineering, The Ohio State University, Columbus, OH USA

**Keywords:** Metagenomics, Microbial ecology

## Abstract

Our knowledge of viral sequence space has exploded with advancing sequencing technologies and large-scale sampling and analytical efforts. Though archaea are important and abundant prokaryotes in many systems, our knowledge of archaeal viruses outside of extreme environments is limited. This largely stems from the lack of a robust, high-throughput, and systematic way to distinguish between bacterial and archaeal viruses in datasets of curated viruses. Here we upgrade our prior text-based tool (MArVD) via training and testing a random forest machine learning algorithm against a newly curated dataset of archaeal viruses. After optimization, MArVD2 presented a significant improvement over its predecessor in terms of scalability, usability, and flexibility, and will allow user-defined custom training datasets as archaeal virus discovery progresses. Benchmarking showed that a model trained with viral sequences from the hypersaline, marine, and hot spring environments correctly classified 85% of the archaeal viruses with a false detection rate below 2% using a random forest prediction threshold of 80% in a separate benchmarking dataset from the same habitats.

## Introduction

Earth’s nutrient and energy cycles are powered by tiny microbial engines [1]. While bacteria are more commonly studied, there is growing recognition that archaea are also critical [2, 3]. For example, archaea can comprise nearly half of the microbial community in the mesopelagic ocean [2]. Here, the Nitrososphaeria (formerly Thaumarchaeota) are the primary ammonia oxidizers, contributing to global greenhouse gas emissions (N_2_O) and accounting for the majority of fixed nitrogen loss below the photic zone [3–5]. In recent decades, a feedback between climate change-driven expansion of low oxygen regions in the mesopelagic ocean, where Nitrososphaeria thrive, and the subsequent increase in greenhouse emissions from these regions, is endangering some of the world’s most productive marine environments [6–8]. In wetlands and permafrost soils, dominant methanogenic Euryarchaeota accounts for up to 40% of the world’s methane production, much of which is further oxidized by co-occurring methanotrophs [9]. This is of particular concern as much of the world’s soil carbon is stored in permafrost regions, which are rapidly transitioning into wetlands as global temperatures increase, thus representing a potential major source of atmospheric methane in the future [10]. Given the abundance and critical biogeochemical roles played by archaea in these and other systems, knowledge of the viruses infecting them is essential for robust ecological assessments and predictive climatic modeling.

Just as bacteria have been well-studied relative to archaea in most natural ecosystems, the same is true of bacteriophages relative to archaeal viruses. Advances in metagenomic sequencing, the ecogenomics sample-to-sequence pipeline, best practices in viral identification [11–14], and analytic platforms such as iVirus that democratized these capabilities [15, 16], have enabled the discovery of hundreds of thousands of bacterial viruses, or phages, from environments around the world [17–20]. These phages are credited with substantially impacting host mortality, horizontal gene transfer, and metabolic reprogramming [21–27], in ways that impact critical ecosystem functions such as global ocean carbon cycling [28]. Thus, our ability to “see” phages is strong, and this has resulted in transformational leaps in our understanding of how phages impact ecosystems.

In contrast, archaeal viruses, which have traditionally been studied in “extreme” environments, such as acidic hot springs, hypersaline ponds, anaerobic sediments, or hydrothermal vents, are severely underrepresented in most global scale metagenome based studies [29–33]. For instance, to date, fewer than 230 marine archaeal viruses have been confidentially identified among multiple metagenomics enabled, or culture-based studies [18, 32, 34–42], while a recent single global oceans survey has revealed over 488 k viral populations, most of which are presumed to be phage [17]. In total, we estimate that there are now well documented genomes or large genome fragments available from fewer than ~380 archaeal viruses, with another 6027 putative archaeal viruses in the IMG/VR-db v3.0, [43] which is a tiny fraction when compared to the hundreds of thousands of population genomes now available for phages [17–20]. An explanation for this may be that novel archaeal virus discovery is largely based on sequence homology searches against public reference databases that are populated by viruses from extreme environments and many archaeal viruses lack homology to these references [44]. These “extreme” archaeal viruses are perhaps not good representatives of those archaeal viruses from relatively non-extreme environments, regardless of the incredible array of morphologies and lifestyles they exhibit [29, 45–48]. Thus, distinguishing bacteriophage from archaeal viruses in datasets from relatively non-extreme environments, using current approaches, remains a challenge, despite the clear genomic and evolutionary differences between phages and archaeal viruses [29, 45, 46, 49–52]. As a consequence, the ecological roles of archaeal viruses in relatively non-extreme environments remain mostly unclear, even while evidence suggests that they may be integral to biogeochemical cycling and host community dynamics [36, 53–55].

The current approach to archaeal virus identification from metagenomic data is based on sequence similarity searches among reference databases, which is severely limiting given the dearth of non-extreme archaeal virus reference genomes. Nevertheless, using this approach we previously developed an annotation-based tool, the Metagenome Archaeal Virus Detector or MArVD, to identify archaeal viruses and used it to discover 43 archaeal viruses from a marine oxygen minimum zone metagenomic dataset [32]. MArVD is now ripe for an update for three reasons: (i) the original tool is reliant on other unsupported software [56], (ii) machine learning has emerged as powerfully enabling in virus ecogenomics for this type of classification task (i.e., DeepVirFinder [57], MARVEL [58], VIBRANT [59], and Virsorter2 [60]), and (iii) there is a growing set of new reference genome data available due to the efforts of several groups manually identifying archaeal viruses from metagenomic sequencing datasets [32–37] and isolate-based datasets [38–42].

Here we introduce and extensively benchmark MArVD2 (Metagenomic Archaeal Virus Detector v2.0) as a machine learning-based upgrade to MArVD that uses curated archaeal virus data from both extreme and non-extreme environments to better leverage the genomic features representative of such archaeal viruses for novel archaeal virus discovery. MArVD2 takes as an input a dataset of viral contigs, pre-identified from tools, such as DeepVirFinder [57], MARVEL [58], VIBRANT [59], and Virsorter2, and returns a list of viruses with their probability of being an archaeal virus.

## Results and discussion

**Fig. 1:**
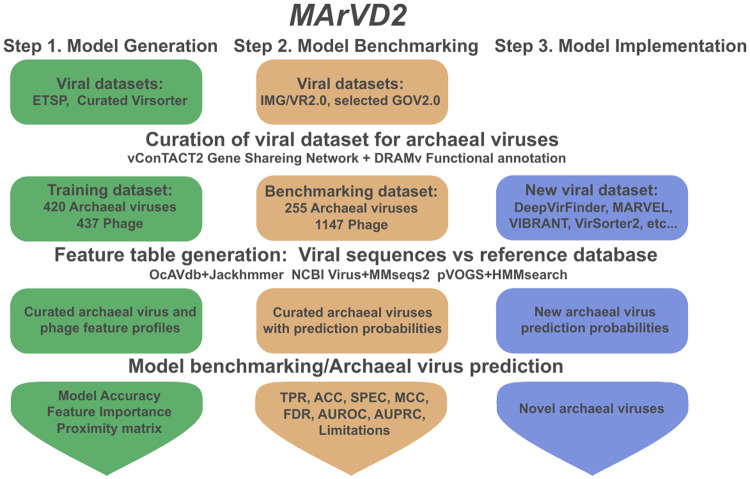
Schematic diagram of the MArVD2 workflow. A representation of the main data processing steps and datasets leveraged in developing and benchmarking MArVD2. MArVD2, as described herein, operates in three modes. First (in green) a training dataset of curated archaeal viruses and phage and several databases of reference archaeal viruses and phage are used to develop a model for archaeal virus identification. Second (in orange) the model is implemented with additional curated archaeal viruses and phage as a benchmarking dataset to evaluate the models’ performance. Third (in blue) a user will supply their own dataset of unclassified dsDNA viruses for archaeal virus prediction using the benchmarked model. See text for dataset description.

MArVD2 is a random forest classifier, implemented in the scikit-learn python package for novel archaeal virus discovery (Fig. 1) [61] where it’s trained and tested with separate datasets of archaeal viruses to best represent its performance in a variety of environments (Fig. 1). Integrating MArVD2 with machine learning introduces several practical and performance improvements over MArVD (version 1) [32], including enhanced usability, with less dependence on other end-user software, increased sensitivity, and greater flexibility to adapt as new archaeal virus databases emerge. MArVD2 retains the very high precision of its predecessor with increased accuracy, enabling robust wide-scale archaeal virus detection from metagenomic datasets.

### Building MArVD2

#### Development of reference, training, and benchmarking archaeal virus datasets

To better represent archaeal viruses from both extreme and relatively non-extreme environments, we first curated several collections of archaeal viruses and phages from a variety of habitats to serve as reference datasets for comparison with the training data, training data for feature generation, and benchmarking datasets for model validation. Reference databases used for genomic feature identification included archaeal virus and phage protein clusters from publicly available repositories (NCBI nr, [62] and pVOGs [63]) and a custom made database of 206 archaeal viruses from the oceans, where new archaeal viruses are being rapidly discovered, curated herein as the OcAVdb or the Ocean Archaeal Virus Database (see below and methods for curation details) [18, 32, 34–39, 41, 42]. The training dataset for the random forest model generation includes 70 non-marine archaeal viruses from the curated VirSorter curated database [64], 350 marine putative archaeal viruses identified from the Eastern Tropical South Pacific (ETSP) [65, 66], and 437 randomly selected bacteriophages from viral RefSeq (v85) [62], the VirSorter curated database [64], and the ETSP dataset (Fig. 1) [65, 66]. Finally, a benchmarking dataset used to examine the performance of MArVD2 under a variety of constraints was comprised of 230 putative archaeal viruses and a random selection of phages with genomes larger than 10 kb from the IMG/VR-db v2.0 [67], along with 25 newly identified marine archaeal viruses from two stations in the Tara Oceans GOV2.0 dataset [17] in environments enriched for archaea (Stations 72_MES and 122_MES). Thus, in addition to those archaeal viruses already available in the NCBI and pVOGs databases, we leverage a total of 881 other archaeal viruses from marine, hypersaline, hot spring, and anoxic environments for reference, training, and benchmarking datasets, establishing a robust base to represent archaeal viruses from both extreme and relatively non-extreme environments.

**Fig. 2:**
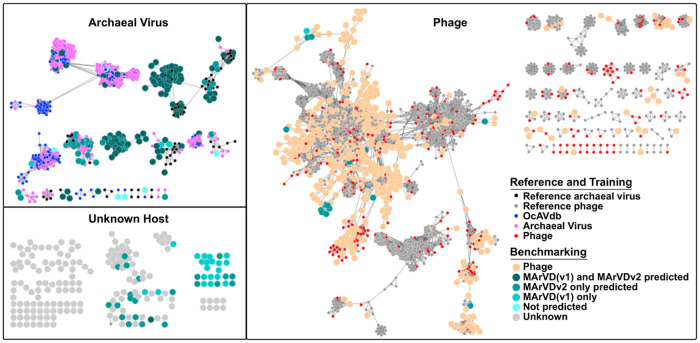
Gene sharing network representation of all training and test viruses used in developing MArVDv2. All sequences used for the development and testing of MArVD2 are included in this network, created by vConTACT2. Reference viruses here include viruses from RefSeq v85 as well as the OcAVdb. Training viruses are those curated from the ETSP and VirSorter datasets as detailed in the text. Benchmarking viruses are those curated from the IMG/VR and GOV2.0 test dataset as detailed in the text. Viruses from the benchmarking datasets are further color coded as either predicted archaeal viruses or phages, from both MArVD and MArVD2. Network modules were grouped according to the inclusion of reference archaeal viruses (archaeal virus), reference phage (phage), or no reference viruses (unknown host).

We next sought to confirm that the collected archaeal viruses for the reference, training, and benchmarking datasets were indeed archaeal viruses. Previously, this was done through meticulous manual screening of gene sharing networks, phylogenetic analysis, sequence homology comparisons, and functional and taxonomic annotations, each of which have revealed that archaeal viruses are distinguishable from phages using these approaches [18, 32, 34–37]. Thus, manual confirmation of archaea as the likely host for the reference archaeal viruses in OcAVdb, the training archaeal viruses, and the benchmarking archaeal viruses was conducted as follows. First, vConTACT2 [68] was used to determine if the archaeal viruses would cluster amongst themselves and separate from phages as has been previously observed (Fig. 2) [32, 49, 50, 52]. Second, manual inspection of the per-gene functional and taxonomic annotations, provided by DRAMv [69], was used to identify archaeal or archaeal virus signatures in each sequence (Supplementary Table 1).

First, network analysis [68] with the OcAVdb references and training archaeal viruses used for model development revealed that the majority of these archaeal viruses clustered with each other and/or other reference archaeal viruses (Fig. 2) (Supplementary Table 2). Out of the 626 archaeal viruses selected for the OcAVdb reference database and the training data, 569 were clustered into 71 viral clusters or VCs (approximately genus-level taxonomic groups [68]) with 45 outliers and 12 singletons. Together these represented 18 network modules (interconnected viral clusters sharing a fraction of their genes [68]) that shared no overlap with phages (Supplementary Table 2). The vConTACT2 network analysis further revealed groupings of archaeal viruses into modules seemingly associated with the Poseidonales or Nitrososphaeria separately, largely corroborating the predicted hosts of these viruses from their respective studies (Supplementary Table 2) [18, 32, 34–37]. Further inspection of the functional annotation of the OcAVdb reference and the training archaeal viruses revealed that on average 17% (stdev 11%) and 27% (stdev 23%) of ORFs per sequence received any annotation, respectively, from KEGG [70] or viral NCBI [62] according to DRAMv [69] (Supplementary Table 1). The training dataset likely received more annotations due to its inclusion of a higher proportion of archaeal viruses from hypersaline environments where archaeal viruses are better characterized [52]. Of the archaeal virus ORFs receiving any annotation in the OcAVdb reference database and the training dataset, 55% (stdev 25%) and 71% (stdev 21%), respectively, affiliated with reference archaea or archaeal viruses. In OcAVdb and training datasets, all but 6 and 2 sequences, respectively, encoded at least one archaeal virus-like ORF, and those that did not generally have a very low proportion of their genes annotated at all (Supplementary Table 1). Notably, early in the curation of OcAVdb, 20 contigs were removed as probable falsely identified archaeal viruses due to a low proportion of genes affiliating with reference archaeal viruses or archaea (only 9 out of 358 total annotated ORFs), and network clustering inconsistent with what is expected of archaeal viruses [32, 49, 50, 52]. Most of these were originally identified by k-mer frequency-based host prediction methods, which can be faulty if the host dataset does not well represent the diversity of the concurrent microbial community (Supplementary Table 2) [71]. As a counterpoint, using the same approach as above, inspecting now the functional annotation of 200 randomly selected phages from the training dataset revealed that on average 70% (stdev 33%) of the phage ORFs were annotated and only 2% (stdev 6%) of these affiliated with reference archaea or archaeal viruses (Supplementary Table 1). While several of these phages were derived from well-curated public databases (NCBI [62] and the Virsorter curated dataset [64]), and represent exceptionally well-annotated viruses, this nevertheless suggests that phages will have a relatively low proportion of ORFs affiliating with archaea or archaeal viruses, relative to genuine archaeal viruses.

Next, manual curation of the benchmarking archaeal viruses used to evaluate the performance of the random forest model once again leverages the network analytics [68] and functional annotations [69]. Network analysis with the benchmarking dataset revealed that 649 of the 1402 total sequences (183 archaeal viruses and 465 phages) clustered into 234 VCs (56 archaeal viruses, 178 phages) with 354 VC outliers (68 archaeal viruses and 287 phages) and 399 singletons (23 archaeal viruses and 376 phage) (Fig. 2, Supplementary Table 2). Out of the 1003 clustered or cluster outlier viruses, 201 archaeal viruses and 582 phages fell into modules with corresponding archaeal viruses or phages from the OcAVdb, NCBI [62], or pVOGs databases [63]. Hereafter, we refer to those archaeal viruses that fell into the same module with reference archaeal viruses as the “verified archaeal viruses”, while those phages that cluster with reference phages as the “verified phage”. Benchmarking archaeal viruses that cluster amongst themselves but with no reference virus, were considered as “putative archaeal viruses”, and those that did not resolve in the network at all are referred to as “archaeal virus singletons”, again with equivalent nomenclature for the phages. Surprisingly, 20 additional IMG/VR phages [67], clustered with archaeal viruses from OcAVdb, NCBI [62], or the VirSorter database [64], possibly indicating a mis-annotation of these viruses in IMG/VR and bringing the total number of verified archaeal viruses up to 221 (Supplementary Table 2).

Functional and taxonomic annotations for the archaeal viruses in the benchmarking dataset revealed that an average of 47% (stdev 33%) of the ORFs per sequence receive an annotation from DRAMv [69]. Out of the ORFs receiving an affiliation, 76% (stdev 30%) matched either archaea or archaeal viruses from the KEGG [70] or NCBI viral databases [62]. Only 10 of these sequences encoded no detectable archaeal signal, 7 of which have <10% ORFs receiving any affiliation. Among the 20 IMG/VR [67] phages predicted as archaeal viruses, 78% (stdev 28%) of the annotated genes matched either archaea or archaeal viruses (Supplementary Table 1).

These analyses, by both gene-sharing networks and genomic functional annotation, indicate that the archaeal viruses in the OcAVdb reference database, the training dataset, and the benchmarking dataset are most likely to be *bona fide* archaeal viruses. Critically, these curated databases drastically expand the available archaeal virus references, which can now be leveraged for more sensitive archaeal virus discovery in non-extreme environments.

#### Development of the random forest archaeal virus classifier—MArVD2

With the now sufficient reference, training, and benchmarking data in hand, we next sought to develop the tool, MArVD2, for more scalable, user-friendly, and sensitive archaeal virus identification by incorporating machine learning. To this end, MArVD2 first populates a feature table consisting of a set of 27 genomic features, which we have predetermined to be informative for archaeal virus identification (Supplementary Table 3), leveraging several databases and tools as follows. First, ORFs are predicted with Prodigal [72], yielding information regarding gene length, gene density, and strand bias. Second, functional and taxonomic annotations are provided by using (i) MMseq2 [73] to search protein-coding regions against viruses in the NCBI nr database [62] (ii) hmmsearch [74] to search against the pVOGs [63] database, and (iii) iterative jackhmmer [74] searches against OcAVdb (Fig. 1). A fivefold cross-validation is then used to recursively identify and retain only the most important features based on the Gini importance index [75] (Supplementary Fig. 1A, B). Finally, MArVD2 then implements the resulting feature table in the development of a random forest machine-learning model for archaeal virus identification, splitting the training data into training and out-of-bag test datasets at a 70:30 ratio, respectively [75].

Evaluation of the random forest model development reveals that MArVD2 exhibits a high degree of performance with the training dataset. Using permutations of the training and out-of-bag test dataset, the F1 score (harmonic mean of the precision and recall, with a score of 1 indicating perfect precision and recall) for the model’s development plateaued at 0.98 with the inclusion of only 8 of the most important features even though all 27 features were identified as contributing to optimal model performance (Supplementary Fig. 1A). This is also reflected by a considerably higher Gini importance score for these 8 features (Supplementary Fig. 1B), indicating that only a subset of the 27 features was required for accurate archaeal virus identification. While building the random forest model (not to be confused with later implementation with the benchmarking dataset), only 19 out of the 857 training sequences (10 archaeal viruses and 9 phages) had inconsistent classifications, according to hierarchical clustering analysis with the random forest proximity matrix (Supplementary Fig. 2). The proximity matrix in this instance is a measure of similarity among the terminal nodes per all decision trees in the random forest model among the given sequences. All 10 of these were viruses of either *Halobacteria*, *Methanobacteria*, or *Thermococci*. Closer inspection of particularly the *Thermococci* sequences revealed this and one other sequence to be pTN2-like plasmids which extensively share replication and regulation genes with other *Thermococcales* viruses [76, 77], further highlighting the value of iterating between model classifications and manual inspection. The main difference between the rest of these proximity outliers and the other training data was a reduced number of hits to the OcAVdb (Mean 3.74 ± 1.72 vs Mean 18.02 ± 20.6, ANOVA *p* = 0.002), the second most important feature in the models’ performance, suggesting that the OcAVdb reference database is not representative of these outliers. Further, out of these 19 poorly characterized proximity outliers, 16 were either singletons or outliers in the vConTACT2 network analysis [68], again indicating that these sequences represent poorly covered sequence space, often with incomplete representatives, in the reference databases (Supplementary Tables 2 and 3). Nevertheless, the high accuracy in classifying the rest of the 410 archaeal viruses compelled us to further evaluate the model’s accuracy on a separate dataset.

### Evaluation of MArVD2s performance

#### Benchmarking MArVD2

**Fig. 3:**
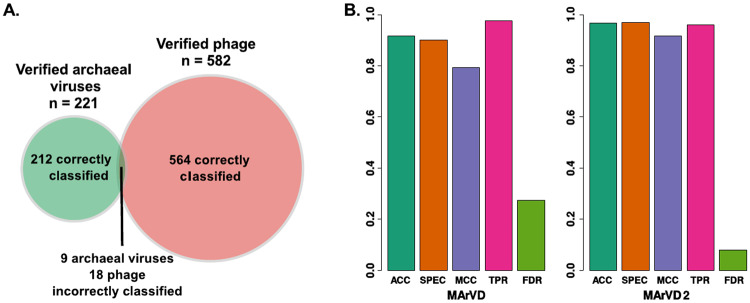
MArVD2 performance and comparison with the original MArVD. **A** Venn diagram representing the number of verified archaeal viruses and phages correctly and incorrectly classified by MArVD2 in the benchmarking dataset. **B** Several performance metrics from the analysis of the benchmarking dataset with either MArVD or MArVDv2. Each metric is recorded with the same proportional units where the higher values indicate better performance, except for FDR where a lower value indicates improved performance. Mathematical definitions for each metric are available in Supplementary Fig. 3.

Random forest classification is drawn from the collective designations of all decision trees per input query, whereby the prediction probability is the proportion of trees agreeing on a particular classification [61]. These prediction probabilities can be interpreted as confidence intervals and provide a high degree of resolution to discern the range of predictions in which MArVD2 will be reliable (Fig. 1) [61]. These confidence metrics are derived from the training set however, and it is good practice to verify these using an independent benchmarking dataset, here including both archaeal viruses and phage from a wide range of environments (see above). Of the 221 verified archaeal viruses in the benchmarking dataset, MArVD2 correctly classified 212, including 13 of the IMG/VR predicted phage that cluster with reference archaeal viruses, while only 9 verified archaeal viruses were missed (Supplementary Table 4). Another 47 putative archaeal viruses were also correctly classified by MArVD2. MArVD2 incorrectly classified only 18 of the 582 verified phages as archaeal viruses (Fig. 3A). Overall MArVD2 had a TPR, ACC, SPEC, MCC, and FDR of 0.96, 0.97, 0.97, 0.92, and 0.08, respectively (Fig. 3B) (See Supplementary Fig. 3 for metric definitions). These results can be compared to what could be expected of a guided homology search without machine learning by considering the same analysis, using the original MArVD (essentially a rule set for archaeal virus identification via homology searches). The original MArVD had a TPR, ACC, SPEC, MCC, and FDR of 0.98, 0.92, 0.90, 0.79 and 0.27, respectively, revealing that MArVD2 had much greater precision but slightly reduced accuracy relative to MArVD (Fig. 3B) [32]. Together, with the fact that the original MArVD is no longer functional due to its reliance on unsupported software, and its relative inflexibility to grow as archaea virus discovery progresses, this makes MArVD2 far superior to its predecessor.

**Fig. 4:**
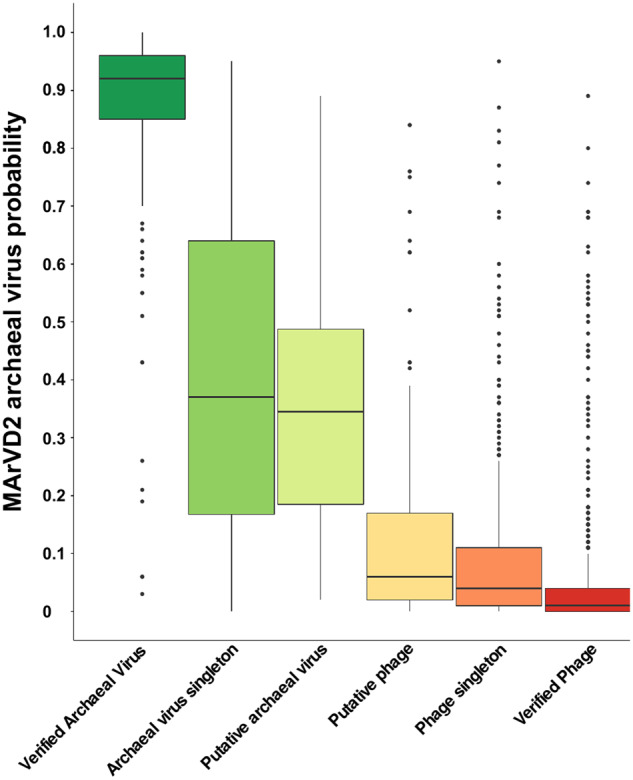
MArVD2 prediction probability of viral populations from the marine, hypersaline, and hot spring viruses, separated into archaeal virus or phage confidence categories. Verified archaeal viruses are those with archaeal or archaeal virus gene homologes and cluster into modules with reference archaeal viruses. Archaeal virus singletons are viral populations suggested to be archaeal viruses by either IMG/VR (*n* = 22) or our manual curation (*n* = 1), but they are not included in any of the vConTACT2 network clusters. Putative archaeal viruses are those suggested to be archaeal viruses by IMG/VR (*n* = 25) or by our manual curation (*n* = 33) and are included in the network, but without references. Equivalent notations apply to the putative (*n* = 144), singleton (*n* = 347), and verified (*n* = 582) phage respectively.

**Fig. 5:**
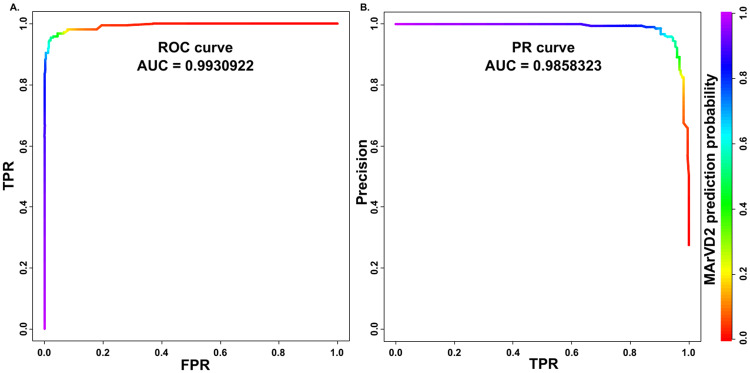
MArVD2 performance on curated benchmarking archaeal viruses and phage. **A** Receiver operating characteristic curve (ROC), plotting the MArVDv2 sensitivity (TPR) versus the FPR. **B** Precision (TP/TP + FP) vs sensitivity (TPR) curve (PR) for MArVDv2 predictions. Data for both (A) and (B) are from the MArVD2 results on the IMG/VR and GOV2.0 benchmarking dataset using only the manually verified phage and archaeal viruses. Quantitative measures of performance for each evaluation are reported as the area under the curve in both A and B where the closer the value to 1, the better the performance. MArVD2 prediction probabilities are reported in the rainbow color gradient.

To better assess the performance of MArVD2 and determine which probability thresholds yield the most optimal results, we evaluated the receiver operating characteristic curve, relative to the prediction probabilities of MArVD2. The verified archaeal viruses from the benchmarking dataset had an average MArVD2 prediction probability of 0.87 (Fig. 4), with a very high area under the receiver operating curve (AUROC) value (0.99) (Fig. 5A). Above this conservative probability threshold, 71% of the verified archaeal viruses (*n* = 157) were identified with only one false positive among the verified phage. Decreasing the probability threshold to 0.80 facilitated the correct identification of 85% of the verified archaeal viruses (*n* = 188) with only 2 false positives among the verified phage. The FPR does not exceed 2% until the MArVD2 probability threshold drops below 0.55, at which point MArVD2 correctly classifies 95% of the true archaeal viruses (*n* = 210) with 13 false positives among the verified phage and another 20 among the putative phage.

When used with unbalanced datasets (i.e., more phage than archaeal viruses), classifiers with a low FPR on benchmark datasets can still yield as many or even more false-positive predictions than true positives, thus rendering the model ineffective. In addition to the detection of archaeal viruses, we also evaluated whether MArVD2 could correctly classify viruses that were *not* archaeal viruses using a precision-recall curve. Here, the area under the precision-recall curve (AUPRC) value again is high (0.99) where the precision of the model does not drop below 98% until sensitivity exceeds 80% (Fig. 5B). Hence the performance of MArVD2 should not be significantly impacted by potentially unbalanced datasets which would include many more phages than archaeal viruses.

Together these analyses indicate that with a permissive prediction probability (we suggest 0.80), MArVD2 will identify most of the archaeal viruses (~85%) from marine, hypersaline, and hot spring environments with very few falsely classified phages.

#### How much genomic information is needed?

Many viral datasets are plagued by short sequences or considerable amounts of microbial contamination which can have major impacts on viral identification and classification [78]. To determine how well MArVD2 would perform on realistic datasets, we split our benchmarking dataset into three test groups to examine the effect of variable dataset size, sequence length, and microbial contamination. The first test dataset included randomly selected sequences from the benchmarking dataset with sequence counts of between 5 and 75% (at 25% intervals) of the original count. The second test dataset includes genome fragments with variable sequence sizes between 1 kb and 10 kb (at 2.5 kb intervals) from the benchmarking dataset. The third test dataset includes varying proportions, between 10 and 75% (at 25% intervals), of randomly selected microbial genomic fragments from IMG/M [79] (equal parts bacteria and archaea) of sizes between 10 kb and 200 kb.

Dataset size in terms of the number of sequences included had a negligible impact on the performance of MArVD2. Across all dataset size fractions (5%, 25%, 50%, 75% number of original sequences) there was minimal variation in TPR, ACC, SPEC, MCC, and FDR relative to the original dataset (average 0.96, 0.97, 0.97, 0.92, 0.8 respectively) (Supplementary Fig. 4).

**Fig. 6:**
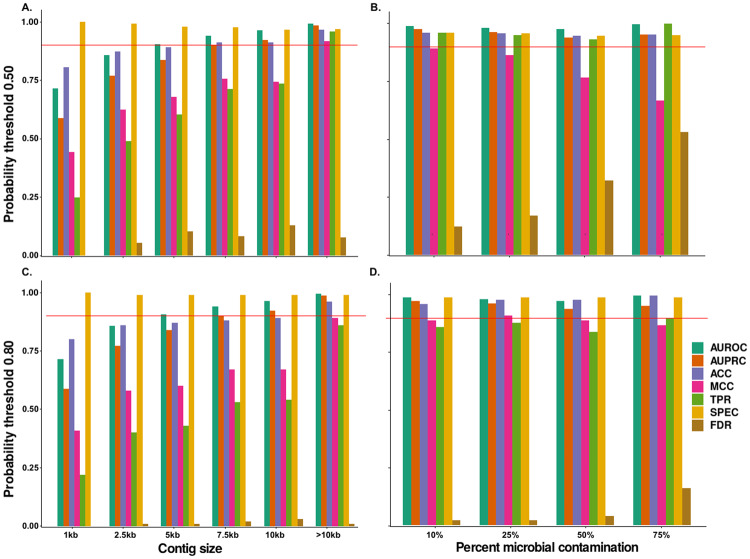
MArVD2 performance with different categories of viral data types. **A** MArVD2 performance relative to contig size using a probability threshold of 0.50. Values were calculated on the IMG/VR and GOV2.0 benchmarking datasets where contig sizes were fragmented into different size categories and randomly selected the same number of input contigs as the original dataset. **B** MArVD2 performance relative to varying proportions of cellular contamination, using a probability threshold of 0.50, with the IMG/VR and GOV2.0 benchmarking datasets supplemented with cellular gene fragments of equal proportions archaea and bacteria of size between 10–200 kb. **C**, **D** These represent the same analysis with an adjusted probability threshold of 0.80, reflecting our recommended threshold. The red line indicates 0.90 where performance is considered acceptable. Performance metrics are described in detail in Supplementary Fig. 3.

Other viral identification machine learning tools such as DeepVirFinder [57], MARVEL [58], VIBRANT [59], and Virsorter2 [60] have reduced performance as virus genome fragment length diminishes. Not surprisingly, we found that MArVD2s performance is impaired on progressively smaller fragments with TPR, ACC, MCC, AUROC, and AUPRC values only exceeding 90% on datasets with contigs>10kbp (Fig. 6A and C). The exception to this was SPEC which remained high and nearly unchanged across the variable sequence size fractioned datasets (1 kb, 2.5 kb, 5 kb, 7.5 kb, 10 kb, >10 kb). Further, the FDR stayed relatively low across all fragment sizes, never exceeding 15% (Fig. 6A, C).

Likewise, increasing amounts of “contaminating” microbial fragments in the viral dataset introduced a higher likelihood of misidentifying a non-viral sequence as an archaeal virus. Even with a 10% inclusion of microbial sequences, MArVD2’s MCC was reduced to less than 90%, while the FDR increased reaching a maximum of 53% with 75% of the input data being microbial. Interestingly, MCC and FDR were the only values influenced by the inclusion of microbial sequences, indicating that the true archaeal viruses were still identified, but that the false positive rate was driven up due to archaeal virus classifications of non-viral sequences (Fig. 6B, D). Notably, when applying the recommended 0.80 prediction probability threshold from above, and using the 75% microbial dataset, the FDR is reduced to 16%, and of the false positives identified above this threshold, all were from Archaea derived from metagenomic datasets.

Pragmatically, this means that for the most optimal performance of MArVD2, we suggest using datasets comprised of contigs no smaller than 10 kb and which have previously been identified as viral by the various available viral identification tools currently available, as well as, an archaeal virus probability threshold of 0.80 (Fig. 6B, D) [57–60].

Beyond these minimal recommendations, we note that the underlying training and test datasets used to develop MArVD2 are predominantly derived from marine, hypersaline, and hot-spring environments. MArVD2 will potentially be ineffective at predicting archaeal viruses from other untested environments or other taxonomic lineages not represented in the current training datasets as it is yet undetermined if these viruses are substantially different from those in the current training datasets. Additionally, it is unclear whether MArVD2 will function with datasets composed of ssDNA viruses, as it has not yet been tested in this capacity. Though currently, this represents a “next frontier” development need, MArVD2 is designed such that it should handle them once appropriate reference genomes become available. In addition, while untested, there is potential that DNA Eukaryotic viruses may be incorrectly identified as archaeal viruses by MArVD2. While the vast majority of dsDNA viruses are thought to infect prokaryotes, we caution users to be aware of these factors.

Contaminating microbial sequences, issues stemming from discerning provirus boundaries, and the potential to miss novel, divergent viral types present considerable challenges to any viral identification effort. These challenges may be exacerbated when further searching for archaeal viruses with relatively unknown sequence space, and which in some cases may share considerable portions of their genome with host elements. These difficulties present potentially unforeseen shortcomings in MArVD2s performance. We strongly encourage the user to carefully examine each putative archaeal virus identified to ensure that the sequence in question is sensibly viral and an archaeal virus.

## Conclusions

Identifying viruses across the Earth’s virosphere is advancing at an astounding pace, with large-scale sequencing and sampling efforts providing new opportunities to see these often hidden, nanoscale ecosystem players. Once identified, the challenge becomes to classify them, where vast inroads have been made with bacterial [68, 80, 81] and eukaryotic viruses [80], but archaeal viruses lag. Here we sought to develop a curated genomic resource and a machine learning-powered tool that will improve our ability to see archaeal viruses in non-extreme environments where archaea themselves have become increasingly recognized as important [3]. Such ability to separate archaeal viruses from other viruses will allow increasing resolution in understanding the ecological interactomes [82, 83] that drive the Earth System.

## Methods

All computation analyses were conducted using the Ohio Supercomputing Center [84], or the National Energy Research Scientific Computation Center, located at the Lawrence Berkeley National Laboratory.

### OcAVdb development

The database of marine archaeal viruses (OcAVdb) was created by collecting all the putative archaeal viruses published from marine metagenomic, single-cell genomes, and viral isolation studies up to 2019 [18, 32, 34–40, 42]. This included a total of 226 archaeal viruses which were further manually curated using vConTACT2 [68] to provide a taxonomic context for each of the putative archaeal viruses, and DRAMv [69] to provided functional annotations. Only those viruses larger than 10 kb which fell into a network module (a collection of related genus scale taxonomic clusters) comprised of only other archaeal viruses, and included archaea or archaeal virus like ORFs were retained in the final database.

### The training and benchmarking dataset development

The training dataset used to develop the MArVD2 random forest model for archaeal virus identification was created by using a combination of public reference databases and databases created by the original MArVD (described below) [32], each vetted by vConTACT2 [68] to include only sequences which fall into the same network module as a reference archaeal virus, and functional and taxonomic annotations affiliating with archaea or archaeal viruses from DRAMv [69]. In total, the training dataset includes 857 virus sequences larger than 10 kb with roughly equal parts archaeal virus and phage. This includes 194 phages from the RefSeq version 85 database [62], 112 phages, and 70 archaeal viruses from the VirSorter database [64] and 131 phages, and 350 archaeal viruses from a published marine environmental virome from the ETSP [65, 66]. Each of these phages and archaeal viruses were selected for inclusion in the training dataset because they cover as much of the taxonomic sequence space as possible according to a network analysis by vConTACT2 and were derived from a variety of environments including hot springs, hypersaline ponds, and the oceans. Training data were implemented in the model creation by Scikit-learn at a ratio of 70 and 30% training and testing datasets [75].

The benchmarking test dataset was created by mining the IMG/VR-db v2.0 [67] for all archaeal viruses from enrichment cultures, the marine environment, hypersaline or alkaline habitats, and thermal hot springs among others. Phages were selected randomly from the same environments with the addition of phages from soils, freshwater, and freshwater sediments. To account for a lack of archaeal viruses from the open ocean in the IMG/VR dataset, an additional 25 putative archaeal viruses from 2 open ocean mesopelagic samples in the Tara oceans GOV2.0 dataset [17] were identified by the original MArVD [32] as described below and included in this test dataset. All viruses in the benchmarking dataset were >10 kbp.

### Re-design of the original MArVD

The original MArVD [32] was recreated as a python 2.7+ script to use the output information from the widely accessible viral identification software VirSorter [64]. This redesigned version of the original MArVD, first uses MetaGeneAnnotator [85] predicted proteins from the VirSorter identified viruses and uses BLASTp [62] to search against the Refseq (version 77) database [62]. Functional and taxonomic annotations are then prescribed in concordance with the highest scoring target sequence with a bitscore >50 and evalue >0.001. These annotations are then integrated into the VirSorter “affi_contigs.csv” gene annotation file retaining the VirSorter derived Pfam [86] designations >40 bitscore and <0.00001 evalue. Using this updated per gene annotation file, MArVD functions exactly as its first inception [32]. Only MArVD category 1 and 2 putative archaeal viruses, corresponding to viruses having over 66 or 50% of their annotated genes affiliating with archaeal viruses respectively, and with bitscore >75 and higher than those for the phage affiliations, were retained as MArVD predicted archaeal viruses. This updated version of MArVD enabled the creation of the new environmental archaeal virus datasets from the ETSP and GOV2.0 datasets needed to train and test MArVD2 as well as allows for a means to compare the performance of MArVD with MArVD2.

### Feature table, databases, and MArVD2 development

Informative features distinguishing archaeal viruses from phages were first identified by generating a feature table containing numerous genome attributes (e.g., average gene length, gene density, strand bias, etc.) (Supplementary Table 3) and combining this with aggregated results from searches against various databases. ORFs were predicted using prodigal [72] with the “-p meta” option. Each of the final set of features were derived either from genomic attributes of the input sequences, an MMseq2 [73] comparison with the NCBI nr [62] database, hmmsearch [74] comparisons against the pVOGs [63] database, or comparison with OcAVdb using jackhmmer [74], each with default parameters. Values and attributes for each feature per input sequence were created and tabulated into a comprehensive feature table which becomes the basis for the random forest model generation [61]. To avoid potential bias introduced in the random forest model by co-correlating features, a co-correlation analysis was performed. Features with greater than 0.95 correlation coefficients were removed. Finally, each virus was designated as archaeal virus or phage and fed into python’s scikit-learn [75] implementation of the random forest model. A manual examination of all archaeal viruses used herein (with the exception of known archaeal viruses in public repositories) is listed in Supplementary Table 1.

MArVD2 first creates the feature table as described above, including only MMseq2 hits with evalues <1e-5, hmmsearch hits with full protein length evalues of <1e-10, and jackhmmer hits with evalues of <1e-5. The MArVD2 random forest model is then built by the python scikit-learn package [75]. To obtain the optimal number of features to create the highest F1 score, recursive feature elimination was used. Features with the lowest Gini importance scores were iteratively removed, with a minimum of five features being retained. fivefold cross-validation of the model’s final accuracy is then calculated using a permuted set of training and out-of-bag test datasets. Multiple additional machine learning algorithms were also tested, but almost always with random forest performing the best. The final random forest model and the preliminary feature table for the training dataset are saved for later implementation with other novel datasets. Re-running the model with new input data will generate a new feature table with archaeal virus or non-archaeal virus predictions and the probabilities associated with those predictions. Hierarchical clustering a visualization of the proximity matrix were conducted using the R packages “vegan” and “pheatmap” [87, 88].

### MArVD2 benchmarking

Using the benchmarking test dataset derived from IMG/VR [67] and the GOV2.0 data [17], we next evaluated the performance of MArVD2 in distinguishing archaeal viruses from phage. The test IMG/VR dataset was first confirmed to be of viral origin by VirSorter [64]. Distinctions between archaeal viruses and phage were next verified by MArVD and confirmed by vConTACT2 [68] network analysis and manual curation of the functional annotations provided by DRAMv [69]. The verifed phage and archaeal virus dataset from IMG/VR were then size fractioned to include contigs of 1 kb, 2.5 kb, 5 kb, 7.5 kb, 10 kb, and >10 kb lengths. For the >10kbp size fraction, a second test dataset with various amounts of microbial sequences was included with equal proportions of bacteria and archaea. Genomic fragments from microbial sequences were randomly selected from the IMG/M [79] database and only included if their size was between 10 kb and 200 kb. Microbial sequences were added at 10, 25, 50, 75, and 95% of the total data. Microbial sequences were ensured not to be viral by use of VirSorter. Dataset size in terms of the number of contigs was also tested with the benchmarking dataset being broken into sets 10, 25, 50, 75, and 95% of the total number of contigs from the original validation dataset.

Sensitivity analysis was then conducted on the unaltered benchmarking dataset and each of the datasets of various size fractions and with various proportions of included cellular sequences. For each dataset, the true positive rate (TPR), specificity (SPEC), accuracy (ACC), Matthews correlation coefficient (MCC) and false detection rate (FDR) was calculated using the R package “EvaluationMeasures“ [89]. The MCC calculation is preferred over an F1 score here because, in practice, environmental datasets will likely have a disproportionate amount of phage to archaeal viruses, so a test that incorporated both the true positives and true negatives will be more informative than one that only includes the true positives. TPR, SPEC, ACC, and MCC were also calculated for the MArVD analysis. AUROC and AUPRC analysis were conducted on each of the datasets using the R package “PRROC” [90]. Visualization of the probability vs host phylum and the statistical assessments were plotted with the R package “pROC” [91], and “gglpot2” [92]. Gene sharing between archaeal viruses and phage was assessed using vConTact2 with default settings by adjusting the “keywords” in the input “proteins.csv”.

### Supplementary information

Supplemental Information

Supplementary Figure 1

Supplementary Figure 2

Supplementary Figure 3

Supplementary Figure 4

Supplementary Figure 5

Supplementary Figure 6

Supplementary Table 1

Supplementary Table 2

Supplementary Table 3

Supplementary Table 4

Supplementary Table 5

## Data Availability

All databases, training data, benchmarking data, OcAVdb, and the random forest model described herein are available on Cyverse at https://de.cyverse.org/data/ds/iplant/home/shared/commons_repo/curated/DeanVik_MArVD2_Apr2022 10.25739/1ttq-2q60 and Zenodo at https://zenodo.org/record/7768113/files/MArVD2_files.tar.gz MArVD2 is available at bitbucket https://bitbucket.org/MAVERICLab/marvd2/ and as a bioconda package at https://anaconda.org/bioconda/marvd2.
