# Supplementary material for: MArVD2: a machine learning enhanced tool to discriminate between archaeal and bacterial viruses in viral datasets
Source: ISME Commun. 2023 Aug 24;3:87. doi: 10.1038/s43705-023-00295-9 (PMC10449787; doi:10.1038/s43705-023-00295-9)
Supplement: Supplementary file 1 — Supplemental Information [file 43705_2023_295_MOESM1_ESM.docx]

**Supplementary information: MArVD2: A machine learning enhanced tool to discriminate between archaeal and bacterial viruses in viral datasets.**

**Authors**

Dean Vik^1,2^, Benjamin Bolduc^1,2^, Simon Roux^3^, Christine Sun^1^, Akbar Adjie Pratama^1^, Mart Krupovic^5^ & Matthew B. Sullivan^1,2,4^

**Affiliations**

1. Department of Microbiology, The Ohio State University, Columbus, OH 43210, USA

2. Center of Microbiome Science, The Ohio State University, Columbus, OH, United States of America

3. DOE Joint Genome Institute, Lawrence Berkeley National Laboratory, Berkeley, CA, USA

4. Department of Civil, Environmental and Geodetic Engineering, The Ohio State University, Columbus, OH, USA

5. MK: Institut Pasteur, Université de Paris, archaeal Virology Unit, F-75015, Paris, France

**381 words main text, 2 figures, 4 citations**

*Gene sharing among archaeal viruses and bacteriophage:*

To evaluate the potential for gene sharing among archaeal viruses and bacteriophages within each habitat (marine, hypersaline, hot spring), we turned to the vConTACT2 ^1^ gene sharing network analyses, leveraging only the training datasets with verified phage and archaeal viruses. Using the vConTACT2 derived protein clusters, we determined the mean number of genes per sequence per habitat that were in a protein cluster found exclusive to archaeal viruses, shared between archaeal viruses and phage or exclusive to phage (Supplementary Figure 5, Supplementary Table 5). Not surprisingly, the highest proportion of genes shared between bacteriophage and archaeal viruses was observed in the relatively non-extreme marine habitat, followed by the hot spring, and hypersaline environments respectively. It is worthwhile to note that the hot spring and hypersaline environments have higher mean proportions of archaeal virus exclusive genes then marine (nearly matching the proportion of shared genes) (Supplementary Figure 5). While not conclusive, it is possible that this reflects an easier archaeal virus identification in more extreme environments, such as the hot spring or hyper-saline habitats with higher relative proportion of archaeal virus exclusive genes.

*Database representation of archaeal viruses:*

To determine whether the reference databases used in model development are sufficiently representative of archaeal viruses (or not) in the marine, hyper-saline, and hot spring environments, we examined the number of genes per benchmarking virus, per habitat, receiving hits from each respective database (Supplementary Figure 6). In all habitats except the hyper-saline, the genes in the false positives receive slightly more hits than the true positives from the pVOGs ^2^ and MMseqs2 ^3^ databases, which are largely enriched for phage. This trend is substantially reversed in the hyper-saline habitat suggesting that the same databases are more representative of the benchmarking hyper-saline archaeal viruses. The marine jackmmer ^4^ database, which is comprised exclusively of archaeal viruses, also yielded substantially more annotations in the true positives than the false positives in all habitats. The pVOGs database was shown the be the most important for feature generation and archaeal virus prediction, and among the true positives in these habitats >50% of the genes per sequence were annotated by this database. Together, these results suggest that the databases used are sufficiently representative of archaeal viruses in these habitats.

**Supplementary references:**

1. Bin Jang, H., Bolduc, B., Zablocki, O., Kuhn, J. H., Roux, S., Adriaenssens, E. M., et al. (2019). Taxonomic assignment of uncultivated prokaryotic virus genomes is enabled by gene-sharing networks. *Nature Biotechnology*, *37*(6), 632–639. https://doi.org/10.1038/s41587-019-0100-8

2. Grazziotin, A. L., Koonin, E. V., & Kristensen, D. M. (2017). Prokaryotic Virus Orthologous Groups (pVOGs): A resource for comparative genomics and protein family annotation. *Nucleic Acids Research*, *45*(Database issue), D491–D498. https://doi.org/10.1093/nar/gkw975

3. Steinegger, M., Söding, J. (2017). MMseqs2 enables sensitive protein sequence searching for the analysis of massive data sets. *Nat Biotechnol 35,* *1026–1028. https://doi.org/10.1038/nbt.3988*

4. Potter, S. C., Luciani, A., Eddy, S. R., Park, Y., Lopez, R., & Finn, R. D. (2018). HMMER web server: 2018 update. *Nucleic Acids Research*, *46*(W1), W200–W204. https://doi.org/10.1093/nar/gky448

**Supplementary Figures**

**Supplementary Figure 1. MArVD2 model feature assessment. A.** Training data is split by 70% training and 30% out-of-bag test and implemented in a 5-fold cross-validation where subsets of the training and test dataset are iterated, such that all of the data are eventually included in both training and test sets. The iterative training and out-of-bag test datasets are then evaluated against the a priori classification of the training data to derive the accuracy of the model (F1 score), relative to the number of features included. The red line indicates the fewest required features for optimal model performance (n=8). **B.** Gini feature importance, measured as the normalized number of decision tree nodes split by a given feature, for each of the features used for model development, and organized in decreasing importance from left to right. “Archaea” here refers to both Archaea and archaeal viruses.

**Supplementary Figure 2.** **MArVD2 model development proximity matrix.** Hierarchical clustering of Euclidean distance between each training viral population determined according to the prediction (archaeal virus or not) prescribed to each population across all possible decision trees in the random forest model. Populations that received incongruous, and thus ambiguous predictions are designated as outliers.

**Supplementary Figure 3. Mathematical definition for each of the performance assessment metrics used in benchmarking MArVD2.** Counts of TP, FP, TN, and FN were derived by careful manual curation of each viral population as detailed in the text using gene content and gene-sharing network affiliation. All measurements were calculated using the R packages “PRROC” or “EvaluationMeasures”.

**Supplementary Figure 4. MArVD2 performance with different dataset sizes.** The IMG/VR and GOV2.0 benchmarking dataset was split into multiple sets including different proportions of the original number of contigs. The red line indicates a value of 0.90 where the model’s performance is considered acceptable. Detailed descriptions of each metric are available in Supplementary Figure 3.

**Supplementary Figure 5. Proportion of ORFs per viral population in protein families that are exclusive to archaeal viruses or phage or shared.** The training and benchmarking datasets were separated by habitat to denote hotspring, hypersaline, or marine archaeal viruses. All proteins were separated according to protein family as determined by vConTACT2. Proteins that did not cluster into a family were grouped as “Not clustered”. All protein families were then distinguished as being found only in archaeal viruses, only in phage, or shared between archaeal viruses and phage. Boxes represent the range, mean and standard deviation of the per contig protein designation counts.

**Supplementary Figure 6. Variation in database representation of true positive and false positive archaeal viruses from different habitats.** Viral populations were separated into their respective environment of origin and into sets of true positives or false positives from the manual curation. Per ORF annotations were derived from the MArVD2’s summary annotation files per database. The amount of ORFs per population in each category that affiliates with references in each database is represented as **A.** the percent of ORFs per contig and **B.** the absolute count of ORFs annotated.

**Supplementary Tables**

**Supplementary Table 1. Functional annotations of select archaeal viruses in the reference, training, and benchmarking dataset.** Each of the reference (only OcAVdb), training, and benchmarking datasets have two tabs “annotations” and “arch_signal”. The first tab “annotations” is provided by DRAMv with additional columns “any-archaea-signal”, “archaea”, and “archvir” to indicate if the given gene hits to any archaea or archaeal viruses, hits archaea, or hits to archaeal viruses respectively. The second tab “arch_signal” describes the counts per contig, of genes receiving no annotation “blank”, hits to non-archaea “no” or hits to archaea “yes” from the DRAM annotations. These counts are then used to calculate the proportion of each sequence annotated, archaea-like and non-archaeal-like. Equivalent tabs are also provided for select phage, including a tab for the IMG phage which we predict as archaeal viruses, and a note for the few contigs missing annotations.

**Supplementary Table 2. vConTACT2 classification.** This table is the “genome_by_genome_overview.csv” file from vConTACT2, with additional columns “dataset” to distinguish between reference, training, and benchmarking datasets, and “category” to distinguish between predicted archaeal viruses and phage.

**Supplementary table 3. Model generation feature table.** This table is the per sequence feature table describing each of the 27 features, the archaeal virus prediction, and whether or not the sequence was included in the proximity matrix, for the training dataset used in model generation.

**Supplementary Table 4. Benchmarking dataset MArVD2 results.** This table is the summarized MArVD2 results for the benchmarking dataset, including results from the manual curation of the benchmarking dataset as described in the text, and the results from the original MArVD.

**Supplementary Table 5. Gene sharing among archaeal viruses and phage.** This table describes the pre-training sequence, the environmental origin of the sequence, and the proportion of genes per sequence encoded by only phage or archaeal viruses or shared among both phage and archaeal viruses.
