# Supplementary figures and images for: MArVD2: a machine learning enhanced tool to discriminate between archaeal and bacterial viruses in viral datasets
Source: ISME Commun. 2023 Aug 24;3:87. doi: 10.1038/s43705-023-00295-9 (PMC10449787; doi:10.1038/s43705-023-00295-9)

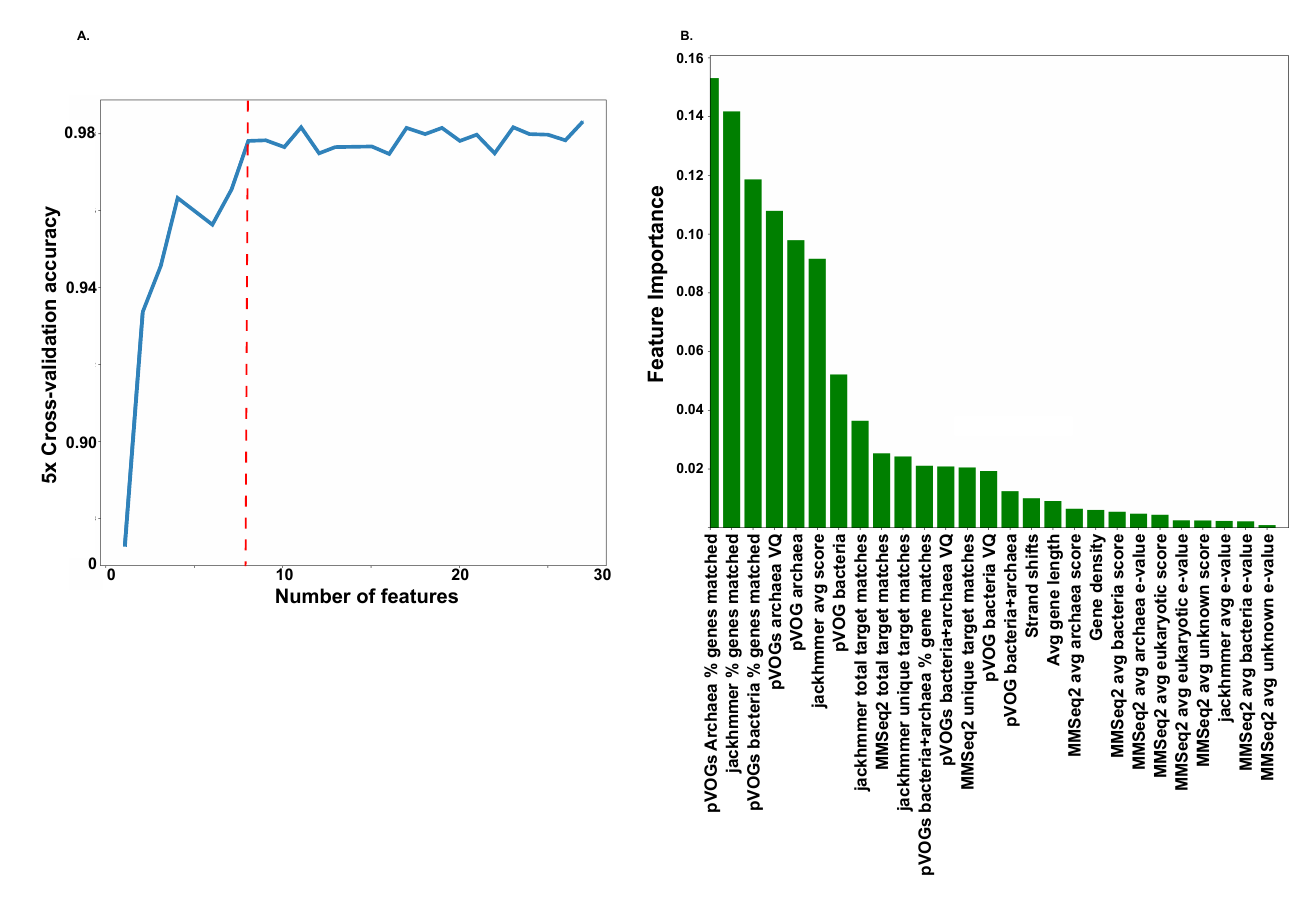

Supplement: Supplementary file 2 — Supplementary Figure 1 [file 43705_2023_295_MOESM2_ESM.tif]

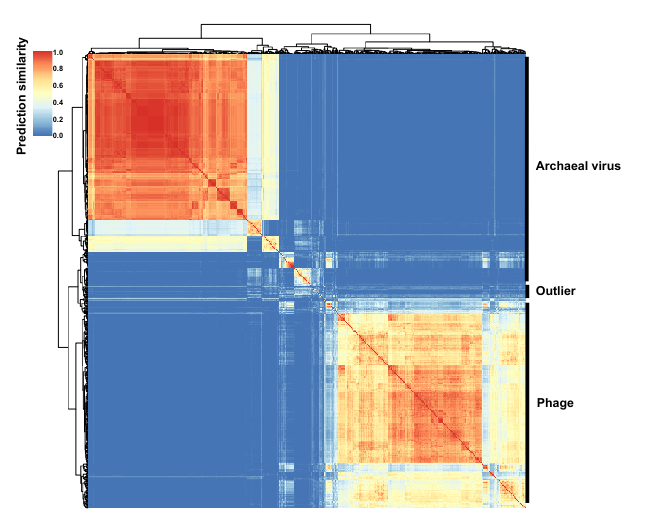

Supplement: Supplementary file 3 — Supplementary Figure 2 [file 43705_2023_295_MOESM3_ESM.tif]

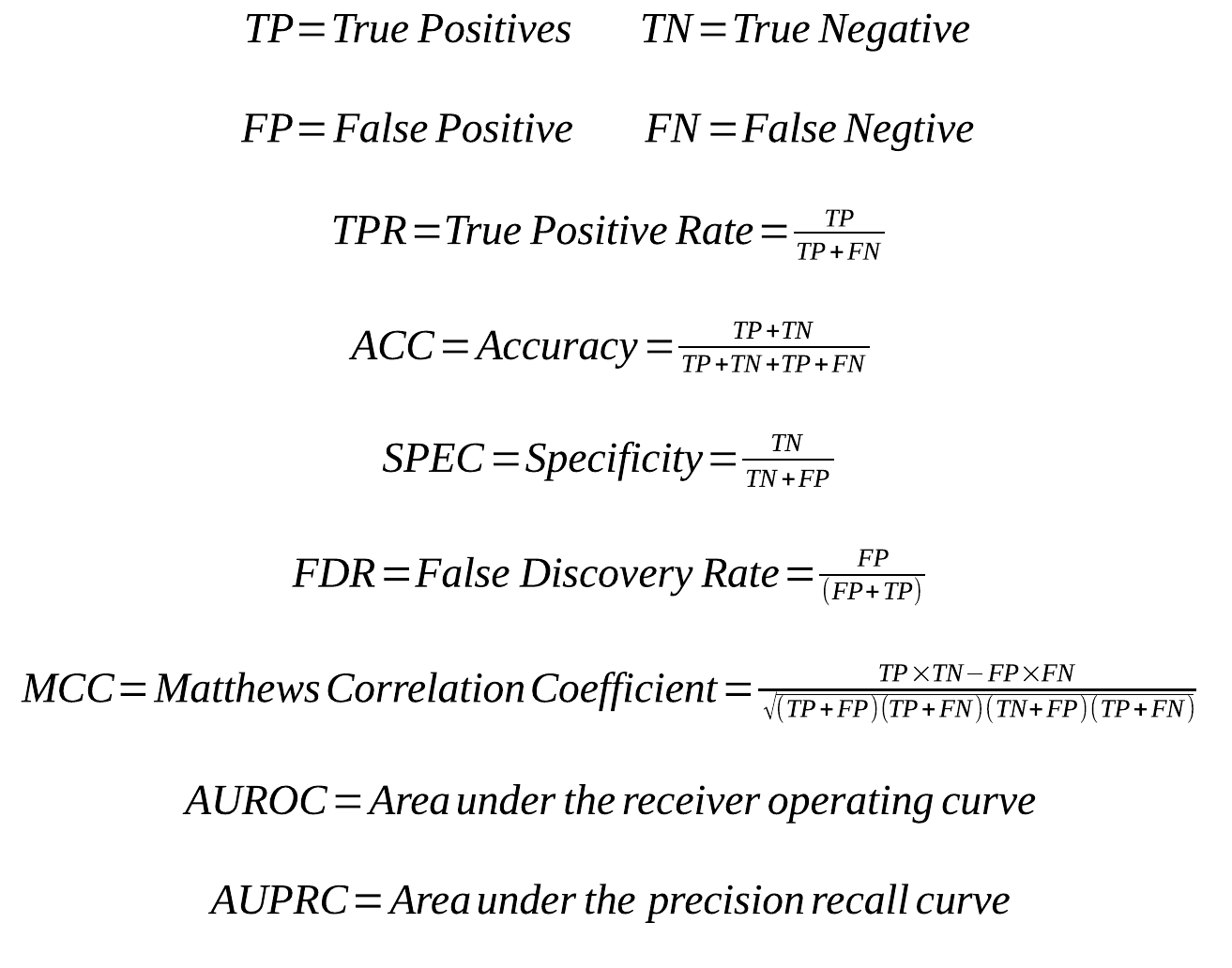

Supplement: Supplementary file 4 — Supplementary Figure 3 [file 43705_2023_295_MOESM4_ESM.tif]

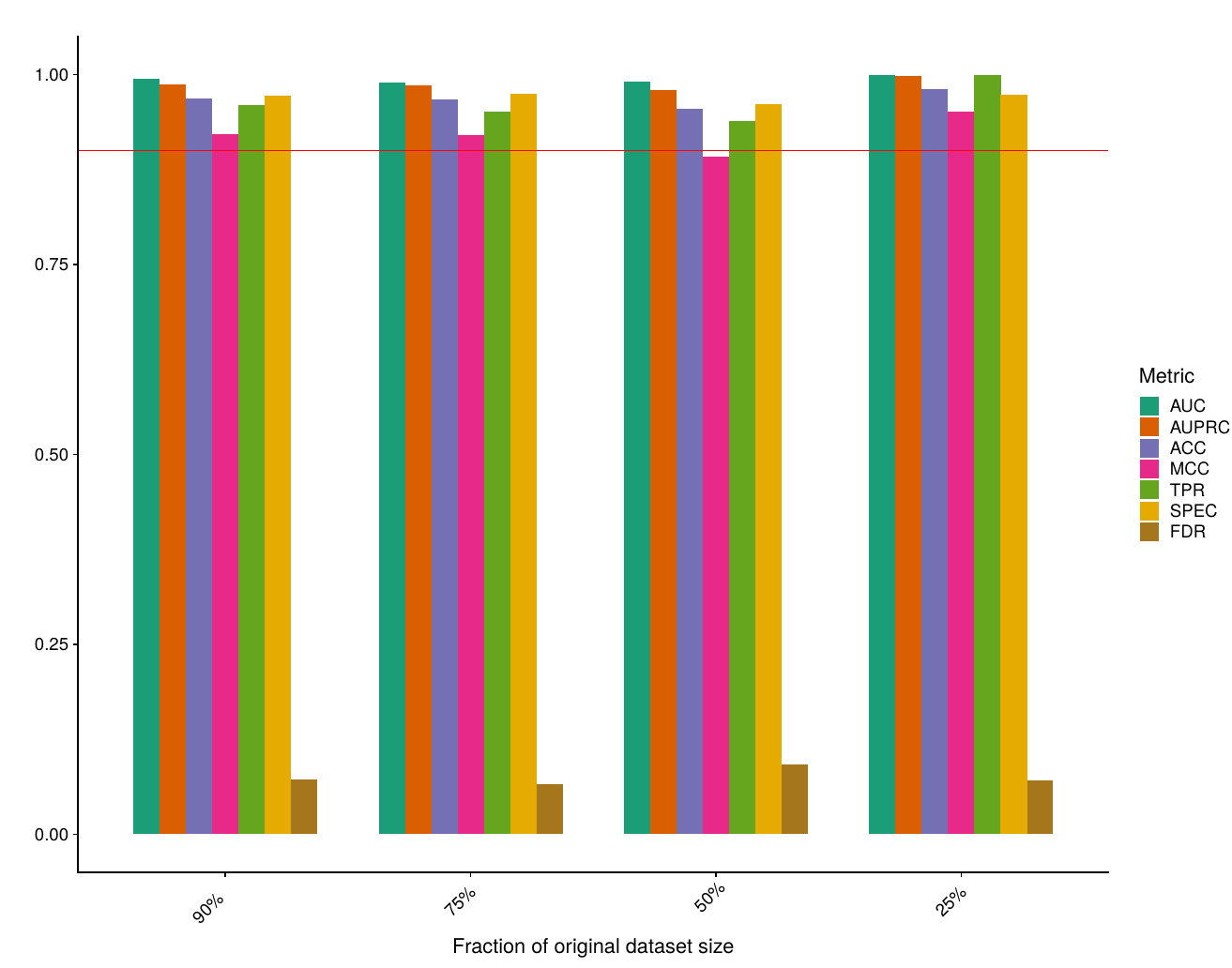

Supplement: Supplementary file 5 — Supplementary Figure 4 [file 43705_2023_295_MOESM5_ESM.tif]

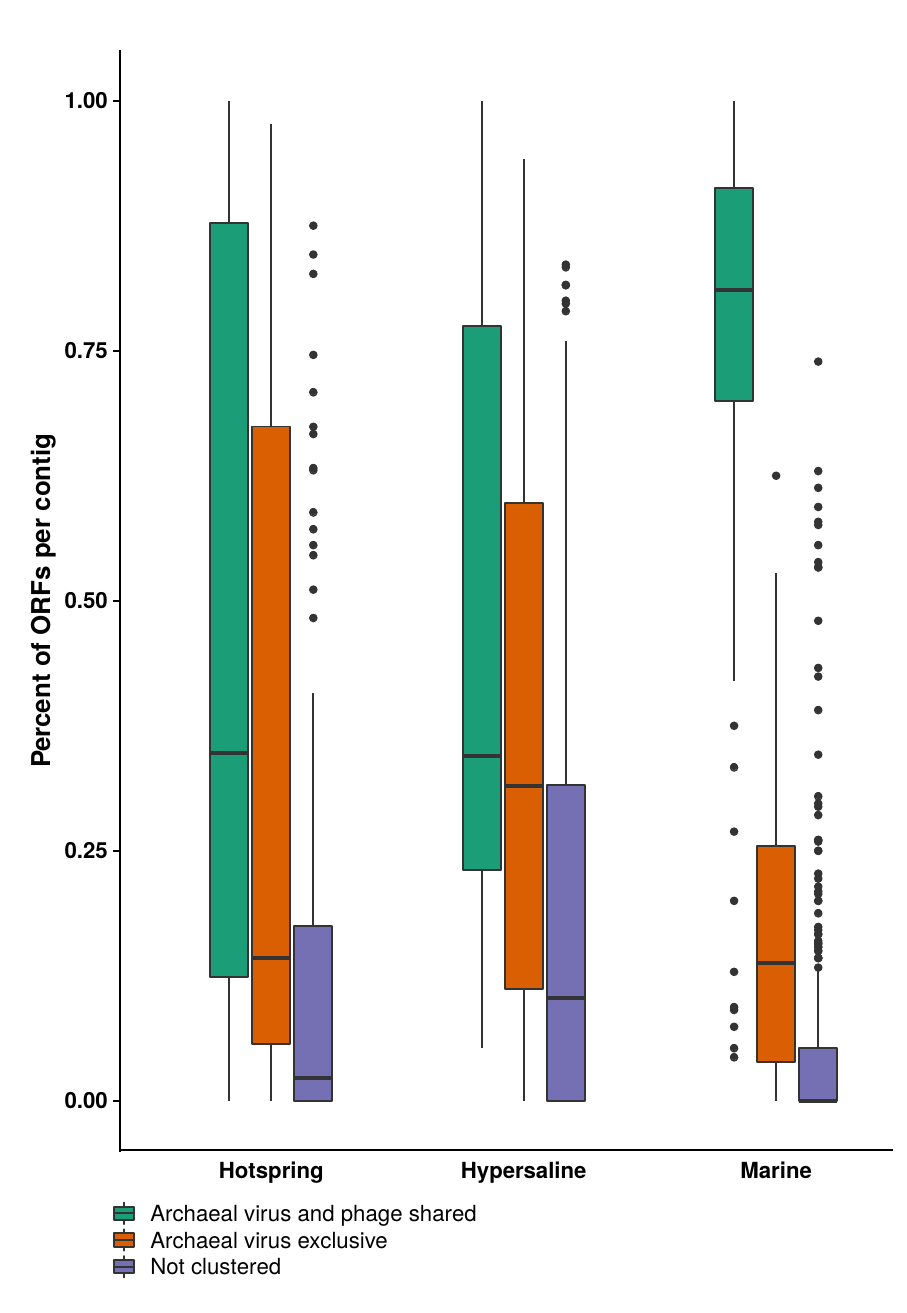

Supplement: Supplementary file 6 — Supplementary Figure 5 [file 43705_2023_295_MOESM6_ESM.tif]

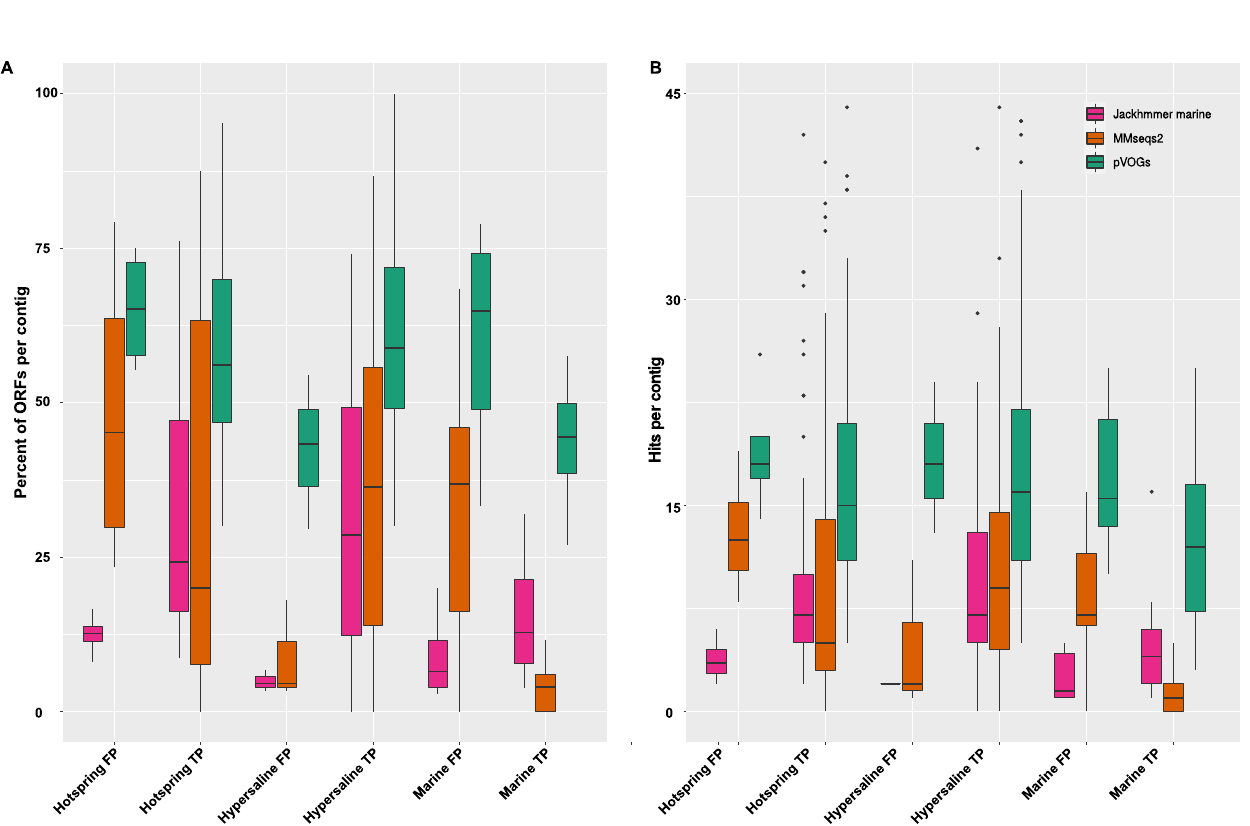

Supplement: Supplementary file 7 — Supplementary Figure 6 [file 43705_2023_295_MOESM7_ESM.tif]
